# 2325: Steroid therapy limits stem cell activation required to enact mucosal healing in inflammatory bowel disease

**DOI:** 10.1017/cts.2017.221

**Published:** 2018-05-10

**Authors:** Evan Brady Lynch, Tatiana Goretsky, Emily Bradford, Tianyan Gao, Terrence Barrett

## Abstract

OBJECTIVES/SPECIFIC AIMS: Intestinal stem cells (ISC) primarily act in the repair of ulcerated epithelium, and their proliferative capacity relies on Wnt/β-catenin signaling. However, the role of GCs on basal epithelial cell signaling has not been fully characterized. The objective of this study was to interrogate a mechanism by which steroids may limit ISC activation. GCs inhibit NFκB signaling, which has been shown to play a role in nuclear β-catenin activation in epithelial cells. We hypothesized that GCs limit Wnt/β-catenin signaling required for ISC activation and epithelial restitution by inhibiting NFκB activation in epithelial cells. METHODS/STUDY POPULATION: To examine the effects of GCs on intestinal epithelial cells, we treated a nontransformed human colonic epithelial cell line (NCM460) with dexamethasone and observed the effects on NFκB and Wnt/β-catenin signaling events. We isolated mouse epithelial cells from the distal colon for stem cell culture as 3D “organoids.” We obtained pure epithelial cell preparations from mucosal biopsies isolated from patients treated at GI clinics at the University of Kentucky Chandler Hospital and VA Medical Center, Lexington. Steroid treated patients with equivalent levels of inflammation, but no mucosal ulceration were used as controls. RESULTS/ANTICIPATED RESULTS: In steroid-treated NCM460 cells, we saw an increase in steroid-responsive genes GILZ and SGK1. We saw a significant decrease in transcripts for Wnt target genes, including Axin2 and cmyc; NFκB target genes, including IFNG and IL6; and the shared NFκB and Wnt pathway co-activator CREBBP, despite unchanged transcript levels for β-catenin (CTNNB1). This data was corroborated in 3D stem cell cultures from cells isolated from mouse colon tissue, which had significant decreases in transcripts for stem cell markers Lgr5 and Ascl2, proliferative markers KI67 and PCNA, and Wnt target Axin2. NCM460s transfected with a lentivirus carrying a TCF/LEF luciferase construct showed a 2.5-fold decrease in TNF-stimulated luciferase activity with dexamethasone treatment. Interestingly, this effect can be rescued by glucocorticoid receptor (GR) blockade with RU-486. Intestinal epithelial cells from patient biopsies showed significant decreases in colitis-induced Axin2, p-LRP6 (a positive marker of Wnt Signaling) and nuclear β-catenin, which correlated with decreased p-p65 protein levels. DISCUSSION/SIGNIFICANCE OF IMPACT: Together, these data suggest that steroid therapy inhibits Wnt/β-catenin signaling at multiple levels, and effects stem cell proliferation in pure stem cell cultures. Decreases in TCF/LEF transcriptional activation (nuclear β-catenin’s DNA binding target) can be reversed with steroid receptor blockade with RU-486, suggesting that a receptor level interaction may be occurring. Interestingly, the required co-activator CBP, shared between NFκB and Wnt pathways, has decreased transcription following steroid treatment, which may provide a mechanism for limited Wnt activation following steroid therapy. Although steroids play a significant role in regulating the amount of inflammatory damage that occurs during IBD treatment, our data suggest that they may be limiting pathways required for effective healing as well.

